# The Robustness Index: Going Beyond Statistical Significance by Quantifying Fragility

**DOI:** 10.7759/cureus.44397

**Published:** 2023-08-30

**Authors:** Thomas F Heston

**Affiliations:** 1 Medical Education and Clinical Sciences, Washington State University Spokane, Spokane, USA; 2 Family Medicine, University of Washington, Spokane, USA

**Keywords:** robustness, percent fragility index, statistical fragility, robustness index, statistical significance

## Abstract

Statistical significance is widely used to evaluate research findings but has limitations around reproducibility. Measures of statistical fragility aim to quantify robustness against violations of assumptions. However, dependence on sample size and single unit changes restricts indices like the unit fragility index and the fragility quotient. The Robustness Index (RI) is proposed to overcome these limitations and quantify fragility independently of the research study's sample size. The RI measures how altering sample size affects significance. For insignificant findings, the sample size is multiplied until significance is reached; the multiplicand is the RI. The sample size is divided for significant research findings until insignificance is reached; the divisor is the RI. Thus, higher RIs indicate greater robustness of insignificant and significant research findings. The RI provides a simple, interpretable metric of fragility. It facilitates comparisons across studies and can potentially increase trust in biomedical research.

## Introduction

Statistical significance, represented by p-values, has been an established cornerstone for evaluating the meaning of research findings since the early 20th century [1,2]. However, multiple pitfalls exist in using and interpreting p-values. The cut-off value of 0.05 is entirely arbitrary and can be misleading [3]. Small sample sizes can result in poorly reproducible results, and the clinical significance of a p-value can have wide variation [4,5].

Statistical fragility refers to the susceptibility of a statistical test to produce different results with only slight changes in the data [6,7]. However, primary fragility measures can be limited by dependence on sample size [8]. This limits comparisons across studies.

The Robustness Index (RI) is proposed to address these limitations and quantify fragility independently of sample size. The RI measures the stability of statistical significance across multiple sample sizes and outcome prevalences. This article explains the rationale, calculation, and interpretation of the RI as a new metric for evaluating statistical fragility.

## Technical report

Current measures of statistical fragility

The Unit Fragility Index

The unit fragility index (UFI) attempts to measure the effect of small changes in outcome upon the statistical significance of the findings. It is applied to research studies that can be categorized into a 2 x 2 contingency table. In the standard 2 x 2 contingency table, the rows represent the experimental condition, and the columns represent the outcome. A common use of the 2 x 2 table is in epidemiology studies examining whether exposure to a toxin is associated with disease (Table 1).

**Table 1:** Standard 2 x 2 Contingency Table a = observed number of subjects exposed with the disease. b = observed number of subjects exposed without the disease. c = observed number of subjects not exposed but with the disease. d = observed number of subjects not exposed without the disease.

Condition	With Disease	Without Disease	Total
Toxin Exposure	a	b	a + b
No Exposure	c	d	c + d
Total	a + c	b + d	a + b + c + d

In Table 1, each box represents an integer. A person is exposed or not and either has the disease or doesn't. So to calculate the UFI, the first rule is that the marginal totals are kept fixed. At the same time, the boxes are incrementally changed by one unit until the statistical significance of the original observed findings is flipped from significant to insignificant or vice versa. Significance can be calculated by Fisher’s exact test or Pearson’s chi-squared test. For example, for a UFI of one, the contingency table would look like Table 2.

**Table 2:** A Unit Fragility Index of One a = observed number of subjects exposed with the disease. b = observed number of subjects exposed without the disease. c = observed number of subjects not exposed but with the disease. d = observed number of subjects not exposed without the disease. If the statistical significance of the outcomes is changed after incrementing by one, then the unit fragility index equals one.

Condition	With Disease	Without Disease	Total
Toxin Exposure	a - 1	b + 1	a + b
No Exposure	c + 1	d - 1	c + d
Total	a + c	b + d	a + b + c + d

The value of the UFI can be altered slightly if the incremental unit change is applied to different outcomes. Therefore, to maintain consistency of the UFI, when the findings are statistically significant, the smallest observed outcome is increased incrementally by one unit, and the other outcomes are simultaneously adjusted by one unit to keep the marginal totals fixed. This is continued until the findings are no longer significant. Alternatively, if the original findings were insignificant, the box with the largest value increases incrementally by 1 until the findings become significant. If an alternative method is required to ensure that no outcome falls below zero, then this method must be explicitly stated when describing the calculation of the UFI.

Researchers can make mistakes in outcome categorization, and subjects are frequently lost to follow-up. The UFI attempts to quantify the significance of these issues. If the UFI is one, then we know that if an error in categorizing just one subject were made, the statistical significance of the study would be flipped. There is no consensus regarding cutoffs for the UFI that would indicate fragility versus robustness. However, a UFI of one or two could reasonably suggest that the research study is highly fragile.

The UFI has limitations because depends upon sample size. For example, the meaning of a one-unit change when the sample size is very large will be less than that of a one-unit change when the sample size is small. This makes the UFI dependent upon the sample size, and therefore it is not possible to compare a UFI of two in a study with a small sample size with a UFI of two when the sample size is very large.

The Fragility Quotient

The fragility quotient (FQ) attempts to overcome the effect of the sample size on the UFI. First, calculate the UFI in the standard fashion. Then, divide the UFI by the sample size (FQ = UFI/sample size). Although the FQ has been used to estimate fragility, there is no consensus on a cutoff value to indicate whether a study is fragile or robust. However, empiric observation suggests that an FQ of 0.03 or less raises a concern about the fragility of the findings and that a closer examination of the data is necessary to determine its meaning.

The Percent Fragility Index

The percent fragility index (PFI) attempts to overcome the effect of the sample size on the UFI and the coarseness of one-unit increments that the UFI utilizes [7]. Instead of incrementally adjusting the categorization of subjects, the PFI looks at the percent change required to flip the significance of the findings. It is applied to the outcome with the highest value, and then each other cell in the 2 x 2 table is adjusted to keep the marginal totals fixed. Table 3 shows how the PFI is applied to a statistically significant study when the number of subjects exposed to the toxin and with the disease is the largest outcome (a).

**Table 3:** Calculation of the Percent Fragility Index a = observed number of subjects exposed with the disease. b = observed number of subjects exposed without the disease. c = observed number of subjects not exposed but with the disease. d = observed number of subjects not exposed without the disease. PFI = percent fragility index

Condition	With Disease	Without Disease	Total
Toxin Exposure	a - (a * PFI)	b + (a * PFI)	a + b
No Exposure	c + (a * PFI)	d - (a * PFI)	c + d
Total	a + c	b + d	a + b + c + d

One important value of the PFI is that its meaning is readily grasped by readers who may not have a statistical background. For example, for a PFI of 1%, readers know that if 1% of the outcomes were miscategorized, then the study's conclusion would be different. While it is important to note that this 1% applies to the largest value, it is more intuitive than the FQ and adjusts for sample size. Like the FQ, a cut-off value for the PFI to determine fragility isn’t established. However, a 5% or less PFI certainly raises concern and skepticism. This means that if there were a 5% or less error in categorizing research subjects, the statistical significance of the study would change.

The robustness index

The RI takes a different approach to fragility in that it does not focus on the miscategorization of data. It does not measure the effect of small changes in categorization upon statistical significance. Instead, it quantifies statistical fragility by looking at how stable the statistical significance is across various sample sizes.

It is well known that larger sample sizes are more likely to be statistically significant and, alternatively, that small sample sizes are less likely to identify a significant finding. This is a basic foundation of statistical power [9].

Calculating the RI is straightforward. It is simply the multiplicand or the divisor required to flip the significance of an observed research finding. It does not need to be an integer. For example, if a 2 x 2 contingency table shows statistically insignificant findings, each box is multiplied until the findings become significant (Table 4).

**Table 4:** Calculation of the Robustness Index for Statistically Insignificant Findings a = observed number of subjects exposed with the disease. b = observed number of subjects exposed without the disease. c = observed number of subjects not exposed but with the disease. d = observed number of subjects not exposed without the disease. RI = robustness index

Condition	With Disease	Without Disease	Total
Toxin Exposure	a * RI	b * RI	(a + b) * RI
No Exposure	c * RI	d * RI	(c + d) * RI
Total	(a + c) * RI	(b + d) * RI	(a + b + c + d) * RI

Similarly, if the original observed findings are statistically significant, then the RI is the divisor, not the multiplicand, required to flip the findings to statistically insignificant. The result is that the RI is always greater than one, regardless of whether the observed findings were significant or insignificant. The RI, as it gets larger, indicates greater robustness of the research findings. The findings are robust and likely reproducible if a study of any size is associated with a large RI. While there is no consensus regarding cutoffs to indicate a study is fragile or robust, an RI of 2 or less suggests that a closer examination of the data is required to determine its meaning.

Take, for example, a recent study examining the effect of peptide receptor radionuclide therapy (PRRT) followed by treatment with somatostatin analogs (SSAs). The researchers wanted to see if sequential therapy with PRRT followed by SSAs was beneficial [10]. In the SSA group, 52 out of 74 had progressive disease, compared to 19 out of 41 in the control group. This difference in outcomes was statistically significant (p = 0.01). The UFI for their findings is 2. This is determined by incrementally adding a one-unit change to the lowest outcome group (19) and seeing when the significance flips from significant to non-significant. This change occurs after adding 2 to the control group and subtracting 2 from the SSA group; if 50 out of 74 in the SSA group had progressive disease, and 21 out of 41 in the control group had progressive disease, then the p-value = 0.08, flipping the significance to insignificant.

While the UFI looks at the effect of adding and subtracting from the outcome groups, the RI looks at changing outcome group sizes by multiplying or dividing. Since the observed findings in this study were statistically significant, the RI is the divisor required to flip the significance to insignificant. The observed findings in this study only become insignificant after the divisor is increased to 1.67, with the resulting outcomes being 31.1 out of 44.3 in the SSA group progressing, compared to 11.4 out of 24.6 in the control group. This results in a p-value of 0.0504. Although the researchers have identified a statistically significant finding, the RI of under 2 suggests that more research is necessary to confirm or refute their findings.

Like the UFI, the RI looks at changes in the p-value; thus, there is no direct formula for its calculation. Instead, it is determined by incremental adjustment to the point where the significance is flipped. It is recommended to determine the RI to 2 decimal places.

## Discussion

Statistical fragility is a problem in medical research that historically has been poorly addressed. Large sample sizes are assumed to correct for fragility; however, even small effect sizes can be statistically significant in large studies. Also, large sample sizes can be prohibitively expensive and thus slow down the progress of scientific research.

The RI proposed in this article offers a valuable new tool for assessing the fragility of research findings. The RI provides a simple and intuitive metric of a study's robustness by accounting for sample size and outcome prevalence, two key factors affecting statistical significance.

Reliance on p-values and statistical significance has come under close scrutiny due to misinterpretation and inherent issues with reproducibility [3,11]. Small changes in assumptions or miscategorized data can dramatically affect p-values. While measures like the UFI attempt to quantify fragility, they are limited by dependence on sample size [6]. The RI attempts to overcome these issues by measuring how changing the sample size affects significance.

The RI calculation progresses as follows: For statistically insignificant findings, the sample size is multiplied until significance is reached. The multiplicand required is the RI. For significant results, the sample is divided until insignificance is reached; the divisor is the RI. A higher RI indicates more robust conclusions. For statistically insignificant findings, an RI of 2 means doubling the sample is needed to change the findings to significant. Alternatively, for statistically significant findings, an RI of 2 means halving the sample size will change the findings to insignificant. 

The RI has several advantages over existing measures. It is simple to calculate and interpret. The RI adjusts for both sample size and prevalence by focusing on how sample size changes affect significance [9]. It is not a percent change in subjects’ classifications like the PFI. This makes it more robust against miscategorizations. It also avoids multiple dependencies like the FQ is based upon.

The RI quantifies robustness on a continuous scale. While no agreed-upon cut-offs designate fragile vs. robust, higher RIs straightforwardly indicate more reliable inferences. An RI under 2 indicates that skepticism is in order. And regardless of sample size, studies with RIs of 5 or greater can confidently be considered robust.

It has become clear that mandatory reporting of a fragility measure in addition to a p-value is necessary for research trials with clinical implications. The RI could serve this role because it makes no assumptions regarding miscategorization and is independent of sample size. If a study reports a p-value of < 0.05 and a RI of > 2, then confidence in the findings is warranted. If the RI is under two, then regardless of the p-value, the results should be viewed with high skepticism.

## Conclusions

The RI provides a valuable new statistical tool to evaluate result fragility. Distilling robustness into a simple, interpretable metric addresses key limitations around sample size dependence. As concern over the reproducibility of p-values grows, the RI offers an important advancement for assessing the validity of scientific findings. The RI has a strong potential to improve fragility evaluation and enhance research reliability. Routine use of the RI could be integrated into reporting standards as a simple yet reliable fragility assessment. Because it is independent of sample size, study registries could include RIs to help assess existing evidence. The RI could also be extended to more complex multivariate analyses. Future fragility research is urgently needed. Thresholds for fragility, similar to the p < 0.05 threshold for significance, need to be empirically tested and then incorporated into routine statistical reporting.
